# Sulforaphane Prevents Angiotensin II-Induced Testicular Cell Death via Activation of NRF2

**DOI:** 10.1155/2017/5374897

**Published:** 2017-01-16

**Authors:** Yonggang Wang, Hao Wu, Ying Xin, Yang Bai, Lili Kong, Yi Tan, Feng Liu, Lu Cai

**Affiliations:** ^1^Department of Urology, China-Japan Union Hospital of Jilin University, 126 Xiantai St, Changchun, Jilin 130033, China; ^2^Pediatric Research Institute, Department of Pediatrics, Wendy L. Novak Diabetes Care Center, University of Louisville, 570 S Preston St, Louisville, KY 40202, USA; ^3^Department of Nephrology, The Second Hospital of Jilin University, 218 Ziqiang St, Changchun, Jilin 130041, China; ^4^The Key Laboratory of Pathobiology, Ministry of Education, The Norman Bethune Medical College, Jilin University, Changchun, Jilin 130021, China; ^5^Cardiovascular Center, The First Hospital of Jilin University, 71 Xinmin St, Changchun, Jilin 130021, China; ^6^Department of Nephrology, The First Hospital of Jilin University, 71 Xinmin St, Changchun, Jilin 130021, China; ^7^Chinese-American Research Institute for Diabetic Complications, School of Pharmaceutical Science, Wenzhou Medical University, Wenzhou, Zhejiang, China; ^8^Department of Nephrology, China-Japan Union Hospital of Jilin University, Changchun, China, 126 Xiantai St, Changchun, Jilin 130033, China

## Abstract

Although angiotensin II (Ang II) was reported to facilitate sperm motility and intratesticular sperm transport, recent findings shed light on the efficacy of Ang II in stimulating inflammatory events in testicular peritubular cells, effect of which may play a role in male infertility. It is still unknown whether Ang II can induce testicular apoptotic cell death, which may be a more direct action of Ang II in male infertility. Therefore, the present study aims to determine whether Ang II can induce testicular apoptotic cell death and whether this action can be prevented by sulforaphane (SFN) via activating nuclear factor (erythroid-derived 2)-like 2 (NRF2), the governor of antioxidant-redox signalling. Eight-week-old male C57BL/6J wild type (WT) and* Nrf2* gene knockout mice were treated with Ang II, in the presence or absence of SFN. In WT mice, SFN activated testicular NRF2 expression and function, along with a marked attenuation in Ang II-induced testicular oxidative stress, inflammation, endoplasmic reticulum stress, and apoptotic cell death. Deletion of the* Nrf2* gene led to a complete abolishment of these efficacies of SFN. The present study indicated that Ang II may result in testicular apoptotic cell death, which can be prevented by SFN via the activation of NRF2.

## 1. Introduction

Infertility affects 6.1 million US couples, representing 10% of reproductive-age adults and 15% of all couples trying to conceive. Half of the time, infertility is the result of an abnormal semen analysis or other male factors [1]. Therefore, there remains an urgent need to identify novel targets and develop novel medicines to prevent male infertility.

Although angiotensin II (Ang II) exerts significant functions in multiple organs and systems [2–4], little is known about Ang II action in male infertility. Both Ang II type 1 and type 2 receptors are found in testis [5], indicating that Ang II may have an important impact on male reproductive function. Previous findings showed that Ang II facilitated human sperm motility [5, 6]. Hence, Ang II may play a beneficial role in male fertility. However, a recent study by Welter et al. showed that Ang II also generated inflammatory events in testicular peritubular cells, in addition to the cell contraction [7]. Consequently, the induction of inflammation by Ang II may exert negative effects in male fertility.

Ang II is found to induce oxidative stress [8, 9]. Previously we reported that Ang II played a critical role in cardiac alcohol-induced cardiac nitrosative damage, cell death, remodelling, and cardiomyopathy [10]. We also found that Ang II activated NADPH oxidase-mediated nitrosative damage to induce pulmonary fibrosis [11]. Notably, oxidative stress contributes to testicular apoptotic cell death [12–16]. Oxidative stress is also known to induce endoplasmic reticulum (ER) stress [17, 18], which has also been demonstrated to play an important role in testicular apoptotic cell death [19–23]. Moreover, a crosstalk has been established between oxidative stress and ER stress [24, 25]. NRF2 controls cellular defence mechanisms against oxidative stress [26] by turning on the transcription of antioxidant genes, such as* Ho1* and* Nqo1* [27, 28]. Notably, NRF2 plays a critical role in prevention of male infertility, since* Nrf2*-null male mice developed infertility in an age-dependent manner [29]. Therefore, NRF2 activation may be a promising strategy to ameliorate Ang II-induced testicular apoptotic cell death.

SFN is a potent activator of NRF2 [30, 31]. We have demonstrated the critical role of NRF2 in SFN protection against diabetes-induced testicular apoptosis [32], diabetic nephropathy [31], and diabetic cardiomyopathy [33]. However, it is unclear whether or how much NRF2 may contribute to the effect of SFN on Ang II-induced testicular injury. The present study aims to answer the following questions: (1) does Ang II induce testicular apoptotic cell death? (2) Does SFN have protective effect on Ang II-induced testicular injury? (3) Does NRF2 contribute to the protective effect of SFN? And if so, how much? To these ends,* Nrf2*-null mice and their WT controls were subjected to Ang II, in the presence or absence of SFN.

## 2. Methods

### 2.1. Animal Treatment

*Nrf2*-null (*Nrf2*^−/−^) mice with C57BL/6J background (WT) were obtained through breeding of homozygote (*Nrf2*^−/−^) with heterozygote (*Nrf2*^+/−^) following the mating system suggested by Jackson Laboratory (Bar Harbor, ME, USA). C57BL/6J male mice (*Nrf2*^+/+^) were also purchased from the Jackson Laboratory. All mice were housed in University of Louisville Research Resources Center at 22°C, on a 12 h light-dark cycle, with free access to standard rodent feed and tap water. The Institutional Animal Care and Use Committee at University of Louisville approved all experimental procedures for these animals, and all procedures complied with the Guide for the Care and Use of Laboratory Animals by the US National Institutes of Health (2011, eighth edition). To test the preventive effect of SFN on Ang II-induced testis injury, as well as the role of NRF2 in SFN action, eight-week-old WT or* Nrf2*-null mice were randomized into the following groups, respectively (*n* = 5 per group): control (Ctrl), SFN-treated control (Ctrl/SFN), Ang II-treated mice (Ang II), and mice treated with Ang II and SFN in combination (Ang II/SFN). Mice received subcutaneous injections of Ang II (Sigma-Aldrich, St Louis, MO, USA, 0.5 mg/Kg) every other day for two months and SFN (Sigma-Aldrich, St Louis, MO, USA, 0.5 mg/Kg) five days each week for three months as previously described [11, 31, 32, 34, 35]. Mice were then killed with their testis and caudae epididymis harvested for analysis. Experimenters of this study were blind to group assignment and outcome assessment.

### 2.2. Sperm Density Assessment

Caudae epididymis from each mouse was placed in 2 mL Earle's balanced salt solution (Sigma-Aldrich, St. Louis, MO, USA) supplemented with 0.1% bovine serum albumin (Sigma-Aldrich). The epididymis was gently teased with a bent needle to release spermatozoa under observation through a stereomicroscope (Olympus). Sperm density was assessed with a haemocytometer and was presented by spermatozoa count per epididymis [36, 37].

### 2.3. Western Blot Analysis

Western blot was performed using testis tissue as described in our previous study [38]. The primary antibodies included anti-3-NT (Millipore, Temecula, CA, USA; 1 : 1,000), anti-4-HNE (Alpha Diagnostic, San Antonio, TX, USA; 1 : 3,000), anti-Actin (Santa Cruz Biotechnology, Dallas, TX, USA, 1 : 2,000), anti-ATF4 (Cell Signaling Technology, Danvers, MA, USA, 1 : 1000), anti-Bax (Cell Signaling Technology, 1 : 1000), anti-Bcl-2 (Santa Cruz Biotechnology, 1 : 2,000), anti-caspase-3 (Cell Signaling Technology, 1 : 1000), anti-caspase-8 (Cell Signaling Technology, 1 : 1000), anti-caspase-12 (Cell Signaling Technology, 1 : 1000), anti-CHOP (Cell Signaling Technology, 1 : 1000), anti-Histone H3 (Santa Cruz Biotechnology; 1 : 500), anti-IL-6 (Cell Signaling Technology, 1 : 1000), anti-NRF2 (Santa Cruz Biotechnology, 1 : 1000), anti-TNF-*α* (Abcam, 1 : 2,000), and anti-VCAM-1 (Santa Cruz Biotechnology, 1 : 500)

### 2.4. Quantitative Reverse Transcription PCR (qPCR)

qPCR was performed as described in our previous studies [39, 40]. Primers for* Ho1* and* Nqo1* were purchased from Life Technologies (Grand Island, NY, USA).

### 2.5. Histological, Immunohistochemical Staining and Terminal Deoxynucleotidyl Transferase-Mediated dUTP Nick End Labeling (TUNEL) Assay

Testis tissues were fixed immediately in 10% buffered formalin solution after harvesting and were embedded in paraffin and sectioned into 5 *μ*m thick sections onto glass slides. The sections were processed for hematoxylin and eosin (H&E) staining. To test the status of testicular cell apoptosis, TUNEL assay was performed as previously described [32].

### 2.6. Isolation of Nuclei

Testicular nuclei were isolated using a nuclei isolation kit (Sigma-Aldrich) as previously described [41]. Briefly, testis tissue (30 mg) from each mouse was homogenised for 45 s in 150 *μ*L of cold lysis buffer containing 0.5 *μ*L of dithiothreitol (DTT) and 0.1% Triton X-100. After that, 300 *μ*L of cold 1.8 mol/L cushion solution (Sucrose cushion solution: sucrose cushion buffer: DTT = 900 : 100 : 1) was added to the lysis solution. The mixture was transferred to a new tube preloaded with 150 *μ*L of 1.8 mol/L sucrose cushion solution followed by centrifugation at 30,000 g for 45 min. The supernatant fraction, containing cytosolic components, was aspirated and the nuclei were visible as a thin pellet at the bottom of the tube.

### 2.7. Statistical Analysis

Five mice per group were studied. The measurements for each group were summarised as means ± SD. Image Quant 5.2 (GE Healthcare Bio-Sciences, Pittsburgh, PA, USA) was used to analyse Western blots. One-way ANOVA was performed for comparisons among different groups, followed by post hoc pairwise comparisons using Tukey's test with Origin 8.6 data analysis and graphing software Lab (OriginLab, Northampton, MA, USA). Differences were significant if *p* < 0.05.

## 3. Results

### 3.1. Deletion of the* Nrf2* Gene Completely Abolished SFN Protection against Ang II-Induced Decrease in Testicular Weight and Spermatozoa Count

**Figure 1:**
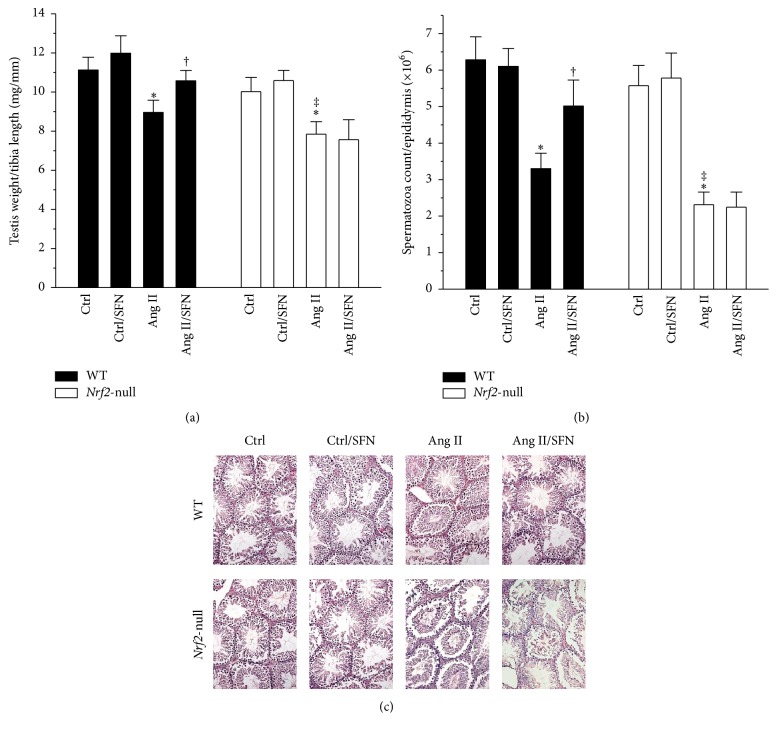
Deletion of the* Nrf2* gene completely abolished SFN protection against Ang II-induced decrease in testicular weight and spermatozoa count. (a) Testis weight to tibia length ratio was calculated after WT and* Nrf2-null* mice were killed. (b) Sperm density assessment was done by performing spermatozoa count. (c) H&E staining was conducted for observation of morphological change. Data are presented as means ± SD (*n* = 5). ^*∗*^*p* < 0.05 versus Ctrl; ^†^*p* < 0.05 versus Ang II. ^‡^*p* < 0.05 versus WT mice treated with Ang II.

*Nrf2*-null mice suffered from a more significant decrease in Ang II-induced testicular weight (Figure 1(a)) and spermatozoa count (Figure 1(b)). WT mice, but not* Nrf2*-null mice, benefited from SFN protection against these injuries (Figures 1(a) and 1(b)). H&E staining showed no significant changes between the groups (Figure 1(c)). These results implicate that testicular apoptotic cell death may play an important role in Ang II-induced testicular weight loss, and NRF2 is required in the protective effect of SFN.

### 3.2. SFN Alleviated Ang II-Induced Testicular Apoptotic Cell Death through the Activation of NRF2

**Figure 2:**
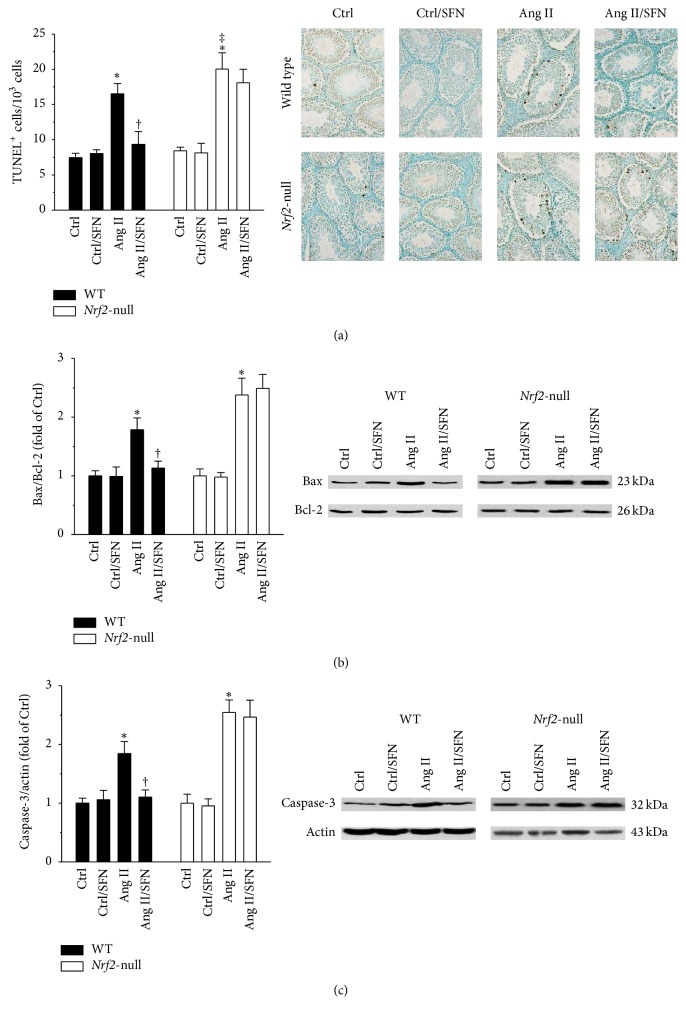
SFN alleviated Ang II-induced testicular apoptotic cell death through the activation of NRF2. (a) TUNEL staining was performed to evaluate the effect of SFN on Ang II-induced testicular apoptotic cell death. Mitochondrial pathway was further evaluated by determining (b) the ratio of Bax to Bcl-2 and (c) the protein level of Caspase-3. For (b) and (c), data were normalised by respective Ctrl and presented as means ± SD (*n* = 5). ^*∗*^*p* < 0.05 versus Ctrl; ^†^*p* < 0.05 versus Ang II; ^‡^*p* < 0.05 versus WT mice treated with Ang II.

Ang II resulted in a marked increase in the number of apoptotic cells in the testis of WT mice (Figure 2(a), left panel). Moreover, this effect of Ang II was more prominent in* Nrf2*-null mice (Figure 2(a), right panel). Mitochondrial pathway was further evaluated by determining the protein levels of Bax, Bcl-2, and caspase-3. Ang II produced a significant increase in the ratio of Bax to Bcl-2 (Figure 2(b)) and caspase-3 protein (Figure 2(c)). SFN prevented these effects of Ang II in WT mice (Figures 2(a)–2(c), left panels). However, deletion of the* Nrf2 *gene abrogated all these protective effects of SFN (Figures 2(a)–2(c), right panels).

### 3.3. ER Stress, But Not Receptor Cell Death Pathway, Was Involved in Ang II-Induced Testicular Injury

**Figure 3:**
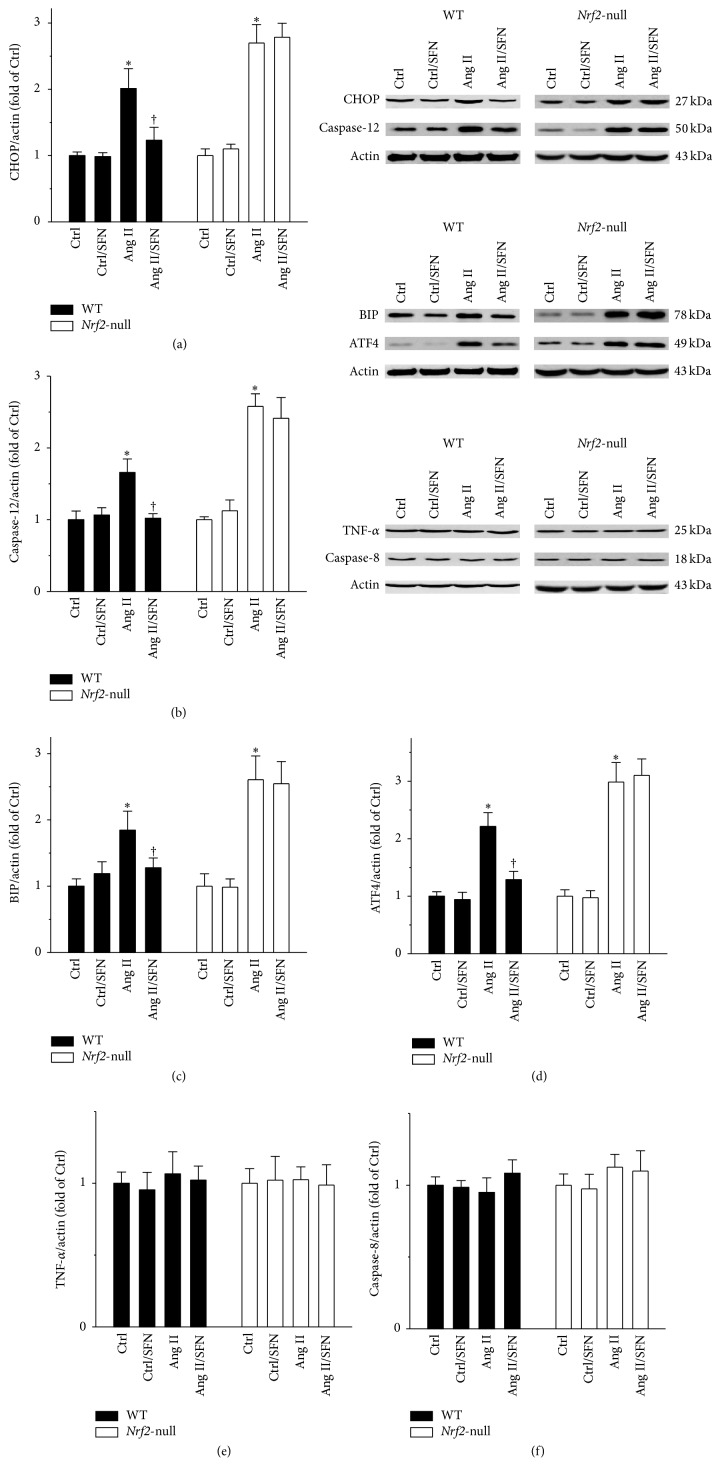
ER stress, but not receptor cell death pathway, was involved in Ang II-induced testicular injury. ER stress was reflected by determining the protein levels of (a) CHOP, (b) caspase-12, (c) BIP, and (d) ATF4. Receptor cell death pathway was also evaluated by determining the protein levels of (e) TNF-*α* and (f) caspase-8. Data were normalised by respective Ctrl and presented as means ± SD (*n* = 5). ^*∗*^*p* < 0.05 versus Ctrl; ^†^*p* < 0.05 versus Ang II.

In the following studies, we defined whether or not ER stress and receptor cell death pathways were involved in Ang II-induced testicular injury. In WT mice, Ang II increased the protein levels of ER stress pathway factors CHOP, caspase-12, BIP, and ATF4, effects of which were markedly prevented by SFN (Figures 3(a)–3(d), left panels). SFN failed to protect the testis from Ang II-induced ER stress in the absence of* Nrf2* (Figures 3(a)–3(d), right panels). No alteration in receptor cell death pathway by either Ang II or SFN was observed, as shown by TNF-*α* and caspase-8 protein levels (Figures 3(e) and 3(f)).

### 3.4. NRF2 Was Required for SFN Amelioration of Ang II-Induced Testicular Inflammation

**Figure 4:**
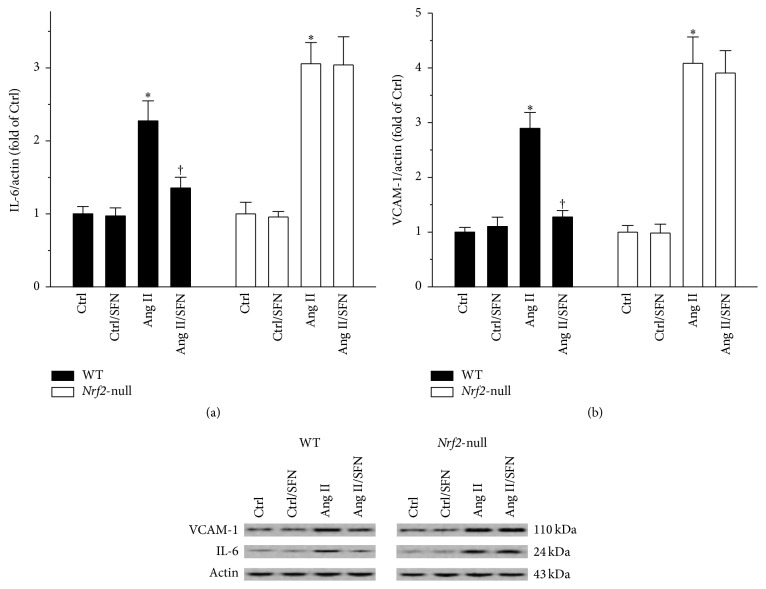
NRF2 was required for SFN amelioration of Ang II-induced testicular inflammation. Testicular inflammatory markers (a) IL-6 and (b) VCAM-1 were measured by Western blot. Data were normalised by respective Ctrl and presented as means ± SD (*n* = 5). ^*∗*^*p* < 0.05 versus Ctrl; ^†^*p* < 0.05 versus Ang II.

Given that Ang II stimulated testicular inflammation in vitro [7], we tested whether or not Ang II could cause testicular inflammation in vivo. Testicular IL-6 and VCAM-1 proteins were elevated by Ang II, effects of which were significantly inhibited by SFN in WT mice, but not in* Nrf2*-null mice (Figures 4(a) and 4(b)).

### 3.5. NRF2 Played a Key Role in SFN Protection against Ang II-Induced Testicular Oxidative Stress

**Figure 5:**
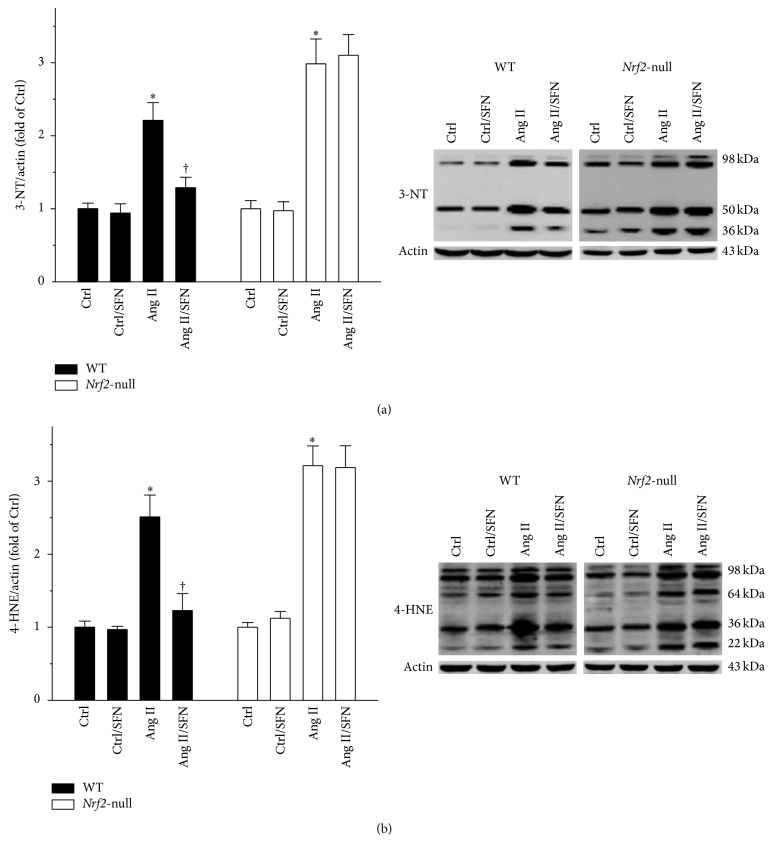
NRF2 played a key role in SFN protection against Ang II-induced testicular oxidative stress. The status of testicular oxidative damage was shown by measuring protein levels of (a) 3-NT and (b) 4-HNE. Data were normalised by respective Ctrl and presented as means ± SD (*n* = 5). ^*∗*^*p* < 0.05 versus Ctrl; ^†^*p* < 0.05 versus Ang II.

The status of testicular oxidative stress was evaluated since NRF2 is known to be the governor of cellular antioxidant activity. 3-NT and 4-HNE, the indicators of nitrosative and oxidative damage, were determined by Western blot. As shown in Figures 5(a) and 5(b), SFN almost completely prevented the Ang II-induced increase in testicular 3-NT and 4-HNE in WT mice (Figures 5(a) and 5(b), left panels), but not in* Nrf2*-null mice (Figures 5(a) and 5(b), right panels).

### 3.6. *Nrf2* Gene Deletion Led to a Complete Loss of SFN Function in Activating Testicular Antioxidant Gene Transcription

**Figure 6:**
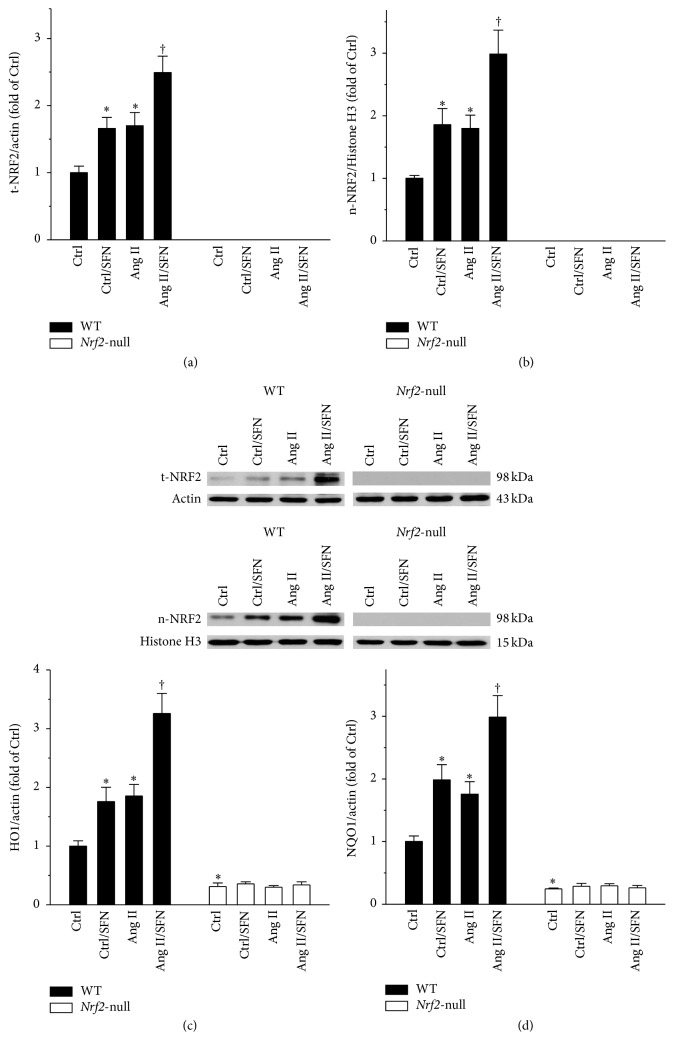
*Nrf2* gene deletion led to a complete loss of SFN function in activating testicular antioxidant gene transcription.* Nrf2* gene expression and function were determined by measuring protein levels of (a) t-NRF2 and (b) n-NRF2, along with (c)* Ho1* and (d)* Nqo1 *mRNAs. t-NRF2, total NRF2; n-NRF2, nuclear NRF2. Data were normalised by WT Ctrl and presented as means ± SD (*n* = 5). ^*∗*^*p* < 0.05 versus Ctrl; ^†^*p* < 0.05 versus Ang II.

NRF2 exerts its function through activation of the transcription of its downstream antioxidant genes. t-NRF2 and n-NRF2 proteins, as well as* Ho1* and* Nqo1* mRNAs, were determined. In WT mice, SFN significantly upregulated testicular t-NRF2 and n-NRF2 proteins (Figures 6(a) and 6(b), left panels), which were not detectable in* Nrf2*-null mice (Figures 6(a) and 6(b), right panels).* Nrf2*-null testis expressed lower* Ho1 *and* Nqo1* mRNAs, as compared to WT testis (Figures 6(c) and 6(d)).* Nrf2* gene deletion disabled the efficacy of SFN in increasing the transcription of* Ho1* and* Nqo1* (Figures 6(c) and 6(d)).

## 4. Discussion

**Figure 7:**
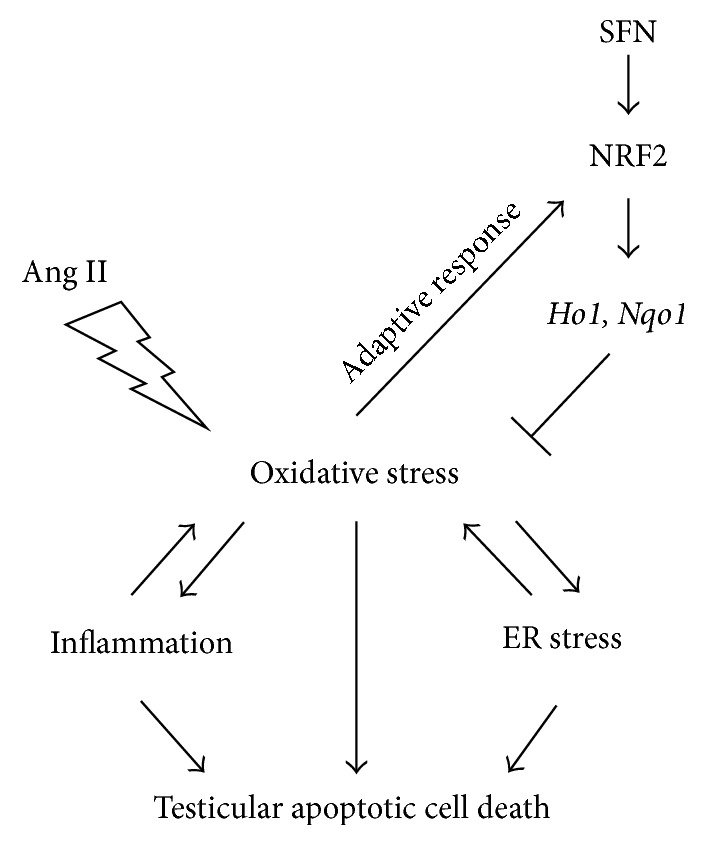
Possible mechanisms for SFN prevention of Ang II-induced testicular apoptotic cell death. Ang II-induced oxidative stress, inflammation, and ER stress contribute to testicular apoptotic cell death. The increased oxidative stress activated NRF2 and the transcription of its downstream target genes* Ho1 *and* Nqo1*, as an adaptive mechanism for defence. Ang II-induced testicular damage could further be alleviated by SFN, via the activation of the NRF2 antioxidant signalling.

The present study explored the protective effect of SFN on Ang II-induced testicular apoptotic cell death. By using* Nrf2*-null mice, NRF2 was found to play a critical role in this protection, since deletion of the* Nrf2* gene led to a complete abolishment of SFN efficacies in the induction of NRF2 downstream targets and in the amelioration of Ang II-induced testicular oxidative damage, ER stress, inflammation, and apoptotic cell death (Figure 7).

In the present study, Ang II increased* Nrf2* expression and function in WT mice (Figures 6(a)–6(d), left panels). The administration of Ang II turned on NRF2 activation as an adaptive response to oxidative stress induced by Ang II. However, the mild increase of NRF2 by Ang II (Figures 6(a) and 6(b), left panels) was not sufficient to block Ang II-induced testicular oxidative damage (Figures 5(a) and 5(b), left panels). The enhanced oxidative damage was almost completely prevented by SFN (Figures 5(a) and 5(b), left panels) via a more significant increase in* Nrf2* expression and function (Figures 6(a)–6(d), left panels). Although the mild increase of NRF2 by Ang II (Figures 6(a) and 6(b), left panels) failed to block Ang II-induced oxidative damage (Figures 5(a) and 5(b), left panels), NRF2 still exerted protective effect, since Ang II produced more severe testicular injuries in* Nrf2*-null mice, as compared to WT mice (Figures 1(a), 1(b), 2(a)–2(c), 3(a)–3(d), 4(a), 4(b), 5(a) and 5(b)).

Ang II is reported to facilitate sperm motility [5, 6]. However, in addition to this beneficial effect of Ang II, Ang II was also found to account for increased inflammation, according to a recent study by Welter et al. [7]. Therefore, a detrimental aspect of Ang II has been unveiled. Inflammation is positively associated with oxidative stress, which plays a key role in testicular cell death [42]. In line with this notion, we found an increase in these indices, along with reduced sperm density and testicular weight in Ang II-treated mice. Our study could be an in vivo support for the previous findings by Welter et al. [7]. Hormesis is defined by a biphasic dose response with specific quantitative features for the amplitude and width of the stimulation [43]. The induction of hormesis by low level stressor agents could rapidly upregulate adaptive processes to repair damage [43, 44]. Therefore, we assume that the discrepancy between the present study and the study by Rossi et al. [6] could possibly be due to the dose difference of Ang II used between the two studies: Ang II concentration used in the previous publication [6] was 0.2 nM. However, we tested toxicological effect of Ang II at 0.5 mg/kg (roughly equal to 0.96 nM, 4.8-fold the value of 0.2 nM), for a long period in mice. In the latter, the toxicity was prominent, as shown by enhanced testicular oxidative damage (Figures 5(a) and 5(b)), inflammation (Figures 4(a) and 4(b)), ER stress (Figures 3(a)–3(d)), apoptosis (Figure 1(b); Figures 2(a)–2(c)), and weight loss (Figure 1(a)). Secondly, results observed in vitro and in vivo may be different since, under the in vivo condition, systemic responses to chronic exposure of Ang II may generate a more complicate outcome in the testis. These important issues will be further explored in the future study.

NRF2 activators have been applied to clinical trials [45, 46]. Although a phase III study of bardoxolone methyl in the treatment of patients with diabetic nephropathy was terminated due to heart complications [47], NRF2 remains a promising drug target, as evidenced by the approval of dimethyl fumarate (also known as BG-12) for use in multiple sclerosis [48]. The possible reasons for the failure of bardoxolone methyl may be the application in an inappropriate stage of disease, the lack of specificity, and interactions between medicines [49, 50]. Although SFN has been tested in many clinical trials [45], none of these was related to testicular diseases. Moreover, very few studies focused on the effect of SFN on testicular diseases in animal models. Thus, there remains an urgent need to test the effect of SFN on various models of testicular diseases, especially the ones with oxidative stress as the main mechanism.

In summary, the present study indicates, for the first time, that Ang II may exert a detrimental function in inducing testicular apoptotic cell death. Other findings suggest that NRF2 is required for SFN protection against Ang II-induced testicular injury.

## Abbreviations

3-NT: 3-Nitrotyrosine
4-HNE: 4-Hydroxynonenal
Actin: *β*-Actin
Ang II: Angiotensin II
ATF4: Activating transcription factor 4
Bax: Bcl-2-associated X protein
Bcl-2: B-cell lymphoma 2
BIP: Binding immunoglobulin protein
CHOP: C/EBP homologous protein
DTT: Dithiothreitol
ER: Endoplasmic reticulum
HO1: Heme oxygenase 1
IL-6: Interleukin 6
NRF2: Nuclear factor (erythroid-derived 2)-like 2
NQO1: NAD(P)H dehydrogenase (quinone 1)
SFN: Sulforaphane
TNF-*α*: Tumor necrosis factor-*α*
VCAM-1: Vascular cell adhesion molecule-1
WT: Wild type.
